# Organization of two kinesins in a two-dimensional microtubule network

**DOI:** 10.1371/journal.pone.0295652

**Published:** 2024-03-13

**Authors:** Jesús M. Bergues, Fernando Falo

**Affiliations:** 1 Universidad San Jorge, Villanueva de Gállego, Zaragoza, Spain; 2 Dpto. de Física de la Materia Condensada, Universidad de Zaragoza, Zaragoza, Spain; 3 Instituto de Biocomputación y Física de Sistemas Complejos, Universidad de Zaragoza, Zaragoza, Spain; University of Toronto, CANADA

## Abstract

In intracellular active transport, molecular motors are responsible for moving biological cargo along networks of microtubules that serve as scaffolds. Cargo dynamics can be modified by different features of microtubule networks such as geometry, density, orientation modifications. Also, the dynamical behaviour of the molecular motors is determined by the microtubule network and by the individual and/or collective action of the motors. For example, unlike single kinesins, the mechanistic behavior of multiple kinesins varies from one experiment to another. However, the reasons for this experimental variability are unknown. Here we show theoretically how non-radial and quasi-radial microtubule architectures modify the collective behavior of two kinesins attached on a cargo. We found out under which structural conditions transport is most efficient and the most likely way in which kinesins are organized in active transport. In addition, with motor activity, mean intermotor distance and motor organization, we determined the character of the collective interaction of the kinesins during transport. Our results demonstrate that two-dimensional microtubule structures promote branching due to crossovers that alter directionality in cargo movement and may provide insight into the collective organization of the motors. Our article offers a perspective to analyze how the two-dimensional network can modify the cargo-motor dynamics for the case in which multiple motors move in different directions as in the case of kinesin and dynein.

## Introduction

Molecular motors are important biological machines for living organisms. Motors attach to the polarized filaments of the cytoskeleton and convert the free energy released by ATP hydrolysis into mechanical work [1–4]. Kinesins, dyneins and myosins are types of processive molecular motors that regulate processes such as active transport and force production [5]. They can move large cargoes across long polymer highways, such as microtubules (MT) or actin bundles [6], by traversing a crowded environment [7]. For example, intracellular transport can be performed by a single kinesin [8]. Experimentally, its mechanical behavior is well characterized [9, 10]. However, when transport is driven by multiple kinesins, variability in mechanical behavior is observed [11–17]. The factors influencing these differences are not known.

Cargo transport of by multiple motors has been modeled considering the symmetrical force-rate separation relationship for a single kinesin [18–22]. However, observed velocities and run times at high forces [23, 24] are not predicted by these models. On the other hand, in reference [25], the authors evidenced an asymmetry in the detachment rate due to the fact that it is higher with assisting forces than with resisting ones. A novel interpretation to this asymmetry was given by reference [26]. When this interpretation is applied to multiple motor assays, it predicts that different experimental geometries result in different load-dependent detachment rates [26].

Cargo transport is influenced by pathways located in a dense network where their structural heterogeneities impose two physical constraints [6, 27, 28]. First, orientations of tracks, as well as branching, results in intersections that might alter the directionally of cargo transport [29]. Second, networks of mixed polarity promote the directionality of motion. This occurs either because in cargo might simultaneously interact with multiple filaments, resulting in a tug-of-war [30], or stochastically detach and reattach to a different track [31, 32]. Thus, all the parameters of MT architecture (density, polarity, filament length, orientation, intersections) can vary between regions of the same cell.

**Fig 1:**
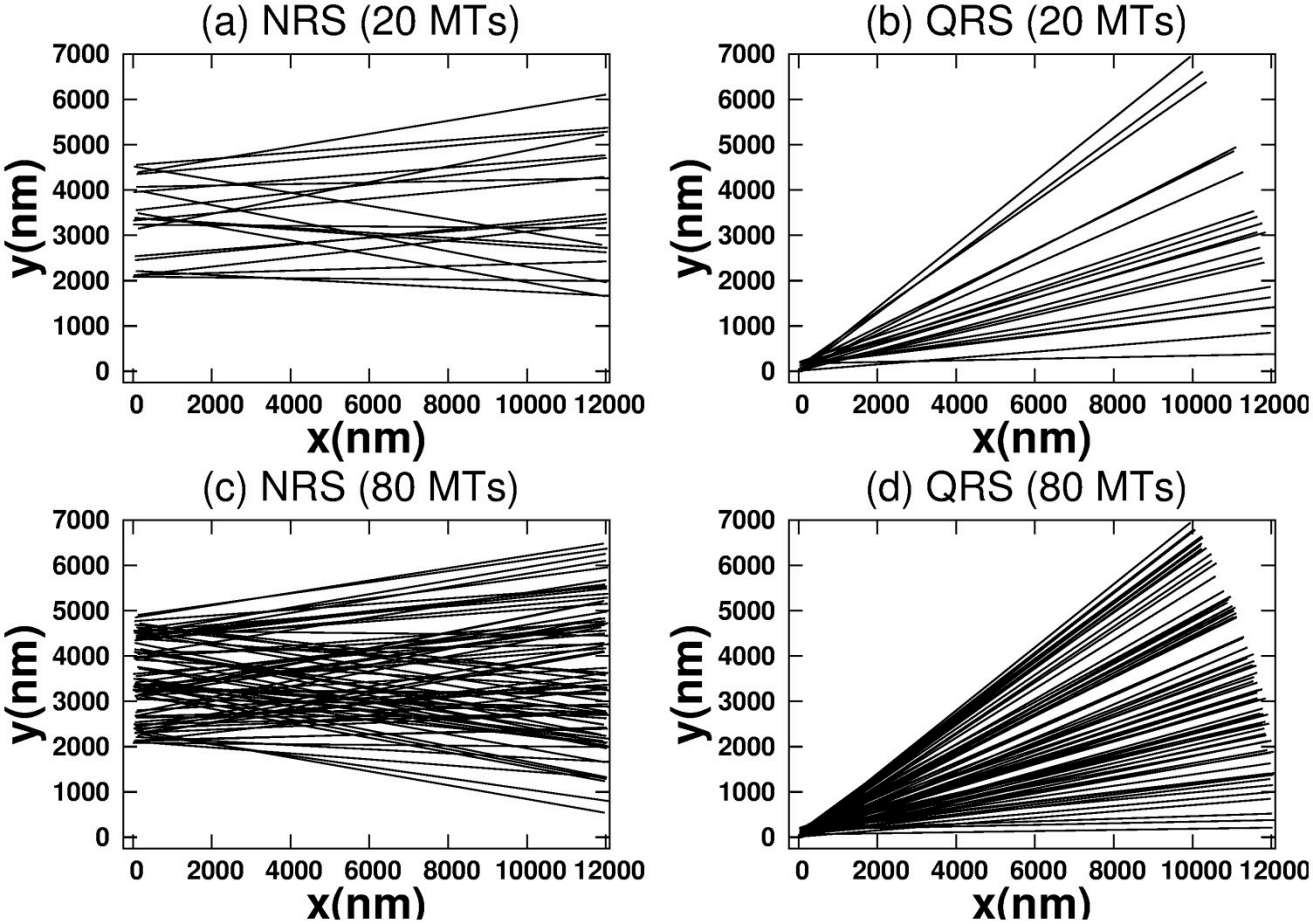
Schematic top view of 2-D networks. (a) and (c) display non-radial structure (NRS) with 20 and 80 MTs, respectively. (b) and (d) show quasi-radial structure (QRS) with 20 and 80 MTs, accordingly.

Inspired by the phenomenology of the transport of cargoes by teams of molecular motor in cells, in this work we develop a minimal model which try to capture the essential traits of the motion of both cargoes and driving motors. Our aim is to elucidate how the organization of motors (kinesins) in the network influences the dynamics of cargo-motor movement and how the indirect motor-motor interactions trough the cargo favors (or not) the transport. Our model is oversimplified since we are considering both motors and cargo as punctual particles and MTs as static linear tracks without volume in a two-dimensional space. These simplifications are a drawback of our model in order to directly compare with experimental results, but it allows us to focus on the interactions between cargo and motors and thus discriminate its influence in the dynamics. Our theoretical framework provides information about cargo transport when it is carried out in a two-dimensional (2-D) network. For this purpose, we consider two types of network configurations: non-radial structure (NRS) and quasi-radial structure (QRS) (see Fig 1). Both structures display different spatial orientations of MTs. The use of a simplified model will solely illustrate the impact of 2-D networks with varying densities of straight and stiff MTs on active transport. Although our minimalistic model limits its scope to realistic situations, our results are a first step to study the cargo-motor dynamics influenced by 3D networks with realistic densities, different orientations and instabilities of MTs. Besides, using a coarse-grained approach [33], we can introduce only a few effective parameters (see below). Thus, it is unnecessary of detailed knowledge on the motion mechanics single motors. Our model provides information about transport efficiency, motor organization and interaction, and critical quantities in 2-D transport (MTs and kinesin numbers). In our study, we first introduce the model and methodology. Then we present our results and discussions. Finally, we draw some conclusions.

## Methods and model

We will consider the Kif5B kinesins as particles that can move along MTs randomly located on a 2-D network as shown in Fig 1. Kinesins can detach from the MT and reattach to them again. On its way, each motor may encounter junctions with other MTs. If the kinesins are not separated from the tracks, they will remain in the same MT or switch to another of the intercepted MT. In each MT, individual motors can occupy discrete positions *r*_*i*_ = *i*Δ*r*, with integer *i* and Δ*r* = 8*nm*. Following reference [34], dynamics of the kinesins is determined by quantities such as, the dwell time *τ*_*D*_(*F*) [9, 10], the forward-backward ratio of jumps *R*(*F*) [9, 10, 35], the detachment rate *P*_det_(*F*), and the attaching rate Π = 5*s*^−1^ [19, 36, 37]. Here, *F* is the force acting on the motor.

According to reference [34], the dwell time can be written as *τ*_*D*_(*F*) = *a*_1_ + *a*_2_[1 + tanh(*a*_3_(*F* − *a*_4_))]. For high ATP concentrations, the values of parameters *a*_*i*_ (*i* = 1, 2, 3, 4) are *a*_1_ = 0.0098*s*, *a*_2_ = 0.07*s*, *a*_3_ = 0.06(*pN*)^−1^ and *a*_4_ = 6*pN*. As in reference [34], *R*(*F*) = *Aexp*(−(*F* log *A*)/*F*_0_) [9, 10, 38], with *A* = 1000 and *F*_0_ = 6*pN* (stall force for a single motor). Both *τ*_*D*_(*F*) and *R*(*F*) permit us to determine the step probability per unit time for forward and backward jumps entering in the Monte Carlo algorithm (see details in the S1 Appendix). Their expressions respectively are *P*_*f*_(*F*) = [*R*(*F*)/(1 + *R*(*F*))]/*τ*_*D*_(*F*) and *P*_*b*_(*F*) = [1/(1 + *R*(*F*))]/*τ*_*D*_(*F*). On the other hand, we consider the detachment rate as *P*_det_(*F*) = exp(−*F*/*F*_*d*_)/(*A*_*d*_*τ*_*D*_(*F*)), with *F*_*d*_ = 3.18 *pN* [34] (detaching force) and *A*_*d*_ = 1 (detachment coefficient). In addition, we considered values of *A*_*d*_ such as 0.002, 0.004, 0.01, 0.2, 1.0, 1.5 and 5.0 to analyze the influence of this factor.

The cargo is modeled as a particle that is transported by two kinesins. This assumption means that this motors are attached to a single spot on the cargo [37, 39]. The cargo performs a continuous overdamped Brownian motion on the 2-D network under the influence of thermal noise and the force exerted by each motor [19]. The cargo is linked to each individual motor by a nonlinear spring [19, 37]. Each motor *i* exerts an attractive force *f*_*i*_ on the cargo (*i* = 1, 2). However, each *f*_*i*_ is non-zero for distances larger than a critical one. The distance between individual motor and cargo is defined by $\Delta_{i}=|{\vec{r}}_{i}-{\vec{r}}_{c}|$; ${\vec{r}}_{c}$ and ${\vec{r}}_{i}$ are the vector positions of the cargo and motor *i*. Each force is defined as *f*_*i*_ = *k*(Δ_*i*_ − *r*_0_) for Δ_*i*_ ≥ *r*_0_ and *f*_*i*_ = 0 if Δ_*i*_ < *r*_0_ and *f*_*i*_ = *k*(Δ_*i*_ + *r*_0_) for Δ_*i*_ ≤ −*r*_0_. The parameters *r*_0_ and *k* are 110 nm (critical distance) and 0.32 pN (*nm*)^−1^, respectively [19, 37]. With the previous assumptions, we consider the dynamical equations for cargo as

$$\begin{array}{c} \gamma \frac{dr_{c}}{dt}=\xi \left(t\right)+\sum_{i=1}^{N}f_{i} \end{array}$$ (1)

here *ξ*(*t*) is the thermal noise satisfying conditions such as 〈*ξ*(*t*)〉 = 0 and 〈*ξ*(*t*)*ξ*(*t*′)〉 = 2*γkTδ*(*t* − *t*′) where *γ* and *T* are the viscous drag and the temperature, respectively. We consider a fixed value *γ* = 9.42 *pN* *s* (*nm*)^−1^ defined by the Stokes formula with a radius of the cargo equal to 0.5*μm* and a viscosity equal to 100 times that of water [19, 37, 40]. The dynamics of each motor is ruled by a Monte Carlo algorithm. When a kinesin detaches from a MT, we assume that the positions of the kinesin and the cargo are the same. Therefore, their coordinates will be the same if the kinesin maintains that condition.

When kinesins move along the same MT, we model the interaction among them by including in the Monte Carlo algorithm a constraint that forbids two motors to be at the same site. So, a motor can only perform a forward (backward) step if the right (left) site is empty (see algorithm details in the S1 Appendix). Besides, motion can occur in different MTs too. So, motor interaction is influenced by the spatial distribution of MTs and the number of motors.

From now on we assume MTs are static straight lines with 12000 *nm* of length. The surface where they are placed can be taken both Cartesian and polar coordinates. Without loss of generality, we can assume a rectangular region with a length and width of 12100 *nm* and 7000 *nm*, respectively. Fig 1(a) and 1(c) display the NRS while Fig 1(b) and 1(d) show the QRS. These networks exhibit different densities, spatial orientations and crossing points for the MTs. For example, the densities of MTs in the vicinity of MT crossing points are different in both networks. On the other hand, all MTs have the negative and positive polarities located in the left and right zones of the networks, respectively. We also call neighboring MTs to those in which the distance to the cargo does not exceed the maximum stretch of the kinesin. All these features modify the movement of kinesins and cargo along the network.

We remark again the scope and limitations of our model. The confinement of kinesins in 2D environments reduces the number of variables involved in cargo-motor dynamics. Thus, we highlight how a certain variable is involved in active transport. For this purpose, we consider the diameters of the MTs to be insignificant and treat them as straight lines. This fact does not take into account the tubular nature of MTs and limits the movement of kinesins to only one protofilament. In addition, it is important to note that MTs exhibit a rigid structure. This characteristic excludes instabilities generated by the alternation between polymerization and depolymerization processes. These processes usually occurs in time scale larger than those we are considering here. Then, using a minimalist model, we aim to find out how the organization of kinesins in a 2D network modifies cargo-motor dynamics in active transport. At the same time, this advantage limits the physiological relevance of the process. However, the information obtained would be useful for establishing procedures to facilitate the analysis of active transport in 3D networks.

Our goal is to analyze how the network structure modifies the cargo-motor dynamics. For this purpose, we will jointly analyze quantities such as: velocity of the cargo (*vc*), histograms of the first cargo passage times (*hfcpt*) to reach 10000 *nm*, motor correlations (*mc*), motor activity (*ma*) and mean intermotor distance (*mid*). All of them are calculated by averaging over 2000 simulations. The acronyms for these quantities are listed in the S1 Appendix.

The *vc* is averaged over the different simulations at each point in time and can be written as $vc=\frac{1}{nsim}\;\sum_{k=1}^{nsim}vca_{k}(t)$. Here, *nsim* is the number of performed simulations and *vca* is the velocity of the cargo in each simulation. This magnitude is obtained by numerical integration of Eq 1.

The *hfcpt* allow us to analyze microscopically when the transport is effective because we can know if every trajectory used by motors for driving the cargo is fast or slow. To classify such speed, we can consider the time used in transport, the time required to initiate the sudden slowing down of the cargo (when the cargo reaches the end of the MT) and and the whole simulation time (25 *s*). Once these parameters are established, we say that a trajectory is fast if it is located up to the third quartile of time (18.8 *s*). At the same time, to characterize the whole transport behavior it is important to take into account other parameters of the distribution of trajectories such as the arithmetic mean, mode, standard deviation, and the coefficient of asymmetry according to Fisher. The values of these quantities are listed in the S1 Appendix.

The *mc* gives the number of motors simultaneously connected to the same or different MTs, indicating how the kinesins are organized in the transport. Mathematically, $mc=\frac{1}{nsim}\;\sum_{k=1}^{nsim}mcor_{k}(t)$. Here, *mcor* are the correlations over time in each simulation. In the correlation over the same MT *mcor* = 1 if both motor are simultaneously attached in the same MT and *mcor* = 0, otherwise. Similarly, for correlation on different MT *mcor* = 1 if both motors are attached to different MTs and *mcor* = 0, otherwise. In the case in which one or both motors are not attached *mcor* = 0. Note that this latest condition makes both correlation non complementary, i.e. their sum is not 1. Thus, the mean values of *mc* are in the range [0, 1].

The *ma* indicates whether the attached motors are exerting force on the cargo, i.e. whether they are active. According to our model, motors are *active* if *f*_*i*_ > 0, otherwise they are *not active*. For our calculations, in each simulation, we assigned the values *mact*_*j*,*k*_ = 1 or *mact*_*j*,*k*_ = 0 to the activity and non-activity states, respectively, of the motor *j* in the simulation *k*. Mathematically, $ma=\frac{1}{\left(2\times nsim\right)}\;\sum_{j=1}^{2}\sum_{k=1}^{nsim}mact_{j,k}(t)$. Here, *mact* stand for the activities over time in each simulation. The mean values of *ma* are in the range [0, 1]. As our system is overdamped (force is proportional to velocity), the profiles of *ma* and *vc* must be similar.

The mean intermotor distance *mid*, along with the *ma* and *mc*, is a quantity that allows us also to characterize the global motor organization in MTs network. Particularly, if *mid* = 0, the kinesins are detached from the MTs. Due to the overdamped nature of the cargo-motor interaction, *mid* and *vc* variations can be related. A *mid* around the distance 110 nm (see Figs 3, 8 and 13) indicates that, for most of the time, just an active motor is attached.

Using the algorithms explained in the S1 Appendix, numerical simulations are performed. Results are obtained from long time realizations that reach out to 25 s with *dt* = 1 × 10^−5^*s* as a time step. At the beginning, all motors are attached to the MTs at random positions in the minus end of MT, either in the same MT or different.

## Results and discussion

### Two kinesins interacting in multiple tracks located in a two-dimensional region

#### Cargo-kinesin dynamics in the NRS and QRS configurations with 80 MTs

**Fig 2:**
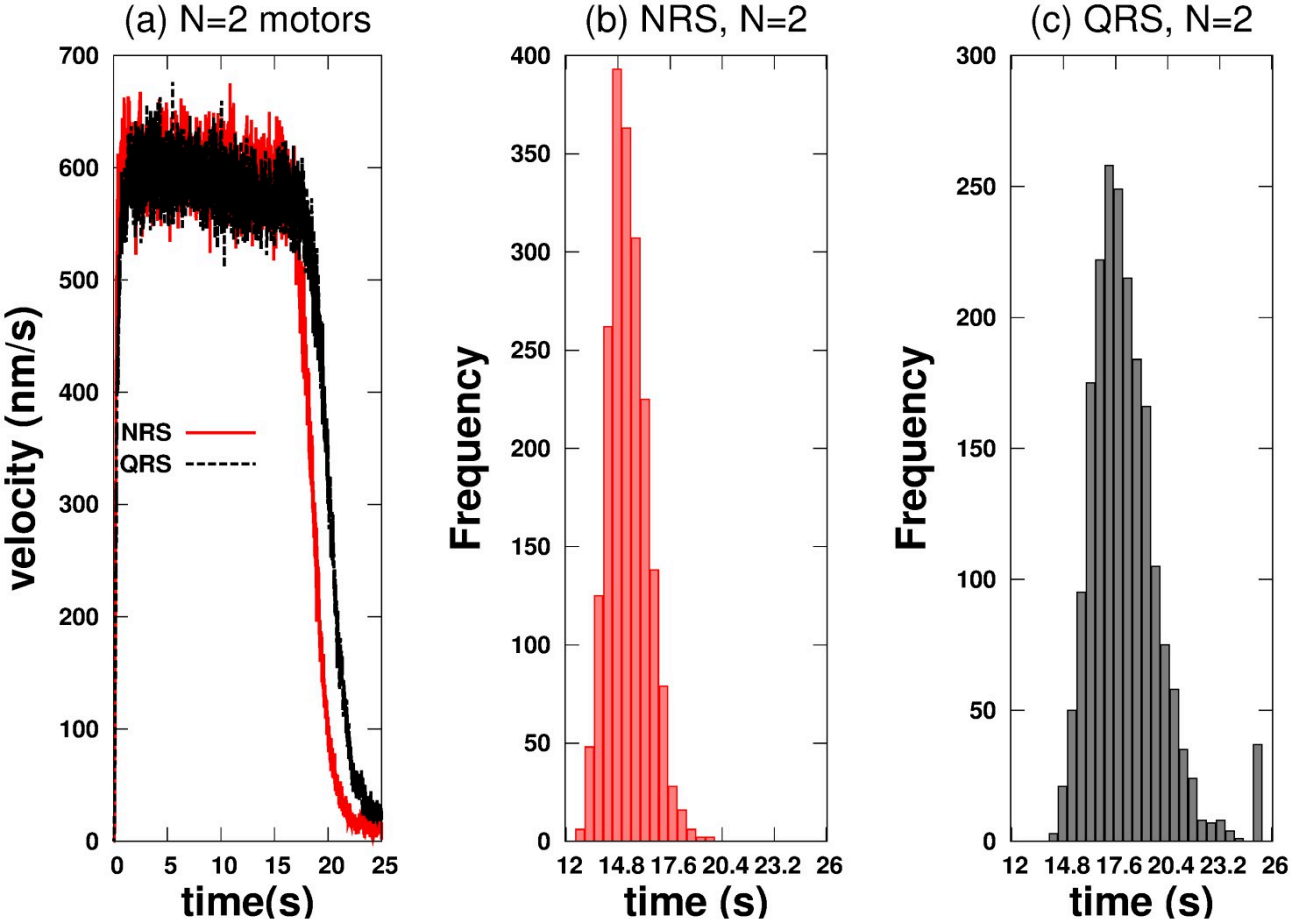
Cargo velocity (*vc*) when driven by two kinesins and histograms of the first passage times (*hfcpt*) to reach a distance of 10000 *nm* on 2-D networks (80 MTs). (a) *vc* on NRS (red color) and QRS (black color). (b) *hfcpt* for NRS. (c) *hfcpt* for QRS.

Fig 2 shows the *vc* and *hfcpt* for the NRS and QRS. In both structures there is a transient of a few seconds during which the kinesins are rearranged (see Fig 2(a)). In this process, the initial positions of the cargo and the kinesins are random, detachment and reattachment events to the MTs are frequent, the distances between the cargo and the motors take on physical significance, and the *vc* changes rapidly. After the transient, the *vc* decreases and fluctuates around values that differ in NRS and QRS. Finally, *vc* suddenly slows down because the kinesins are close to or have reached the end of the MTs.

In Fig 2(a) we can observe that the transport in the NRS configuration is slighly faster than in the QRS one. This behavior is compatible with the histograms in Fig 2(b) and 2(c) and with the values of the statistical variables tabulated in the S1 Appendix. Both histograms show a right skew, with Fisher-skewness coefficients of 0.47 and 1.17 for NRS and QRS, respectively. Therefore, the mean values exceed the mode ones in both configurations. For NRS, the mean is 15.33 *s*, and the mode is 14.5 *s*. Meanwhile, for QRS, the mean and mode are 18.13 *s* and 1.17 *s*, respectively. On the other hand, the standard deviation of 1.04 *s* in the NRS is lower than the 1.85 *s* in the QRS. Based on the given data, it is evident that the rapid trajectories tend to cluster closer to the mean and mode in the NRS than in the QRS. As a result, the cargo velocity is expected to be higher in the NRS.

**Fig 3:**
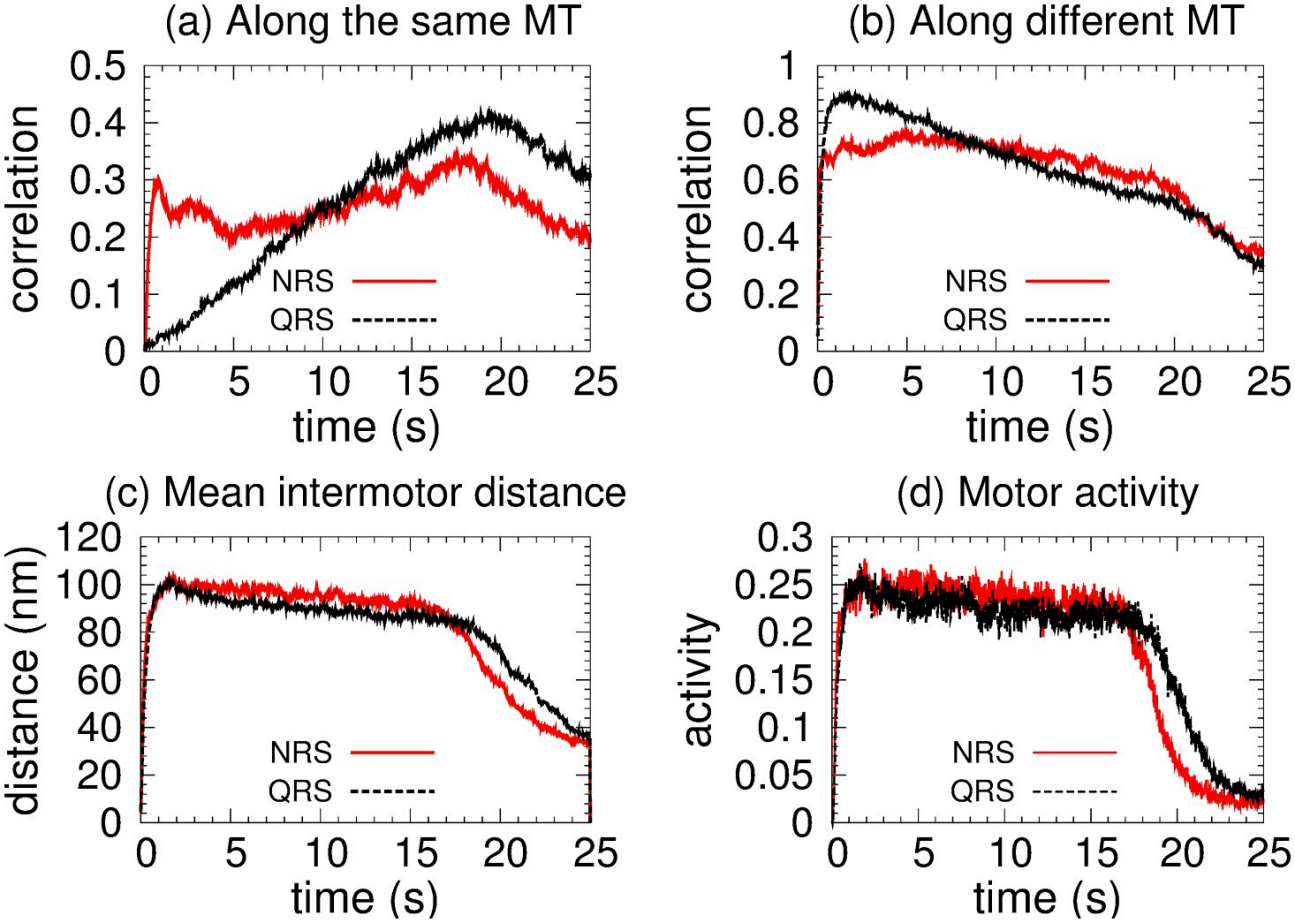
Two kinesins in the NRS (red color) and QRS (black color) network configurations, each with 80 MT. (a) Correlation along the same MT. (b) Correlation along different MTs. (c) Mean intermotor distance. (d) Motor activity.

Fig 3(a) shows a rapid increasing of *mc* in the same MT for NRS and a gradual one for the QRS case, which is compatible with the results of Fig 3(b) of *mc* in different MT. This behavior indicates a rapid organization of the kinesin team (for NRS) which derives in a higher cooperativity in the transport. For the QRS case a higher correlation is achieved but results in lower *vc* due that detach and reattach events are frequent as is reflected in Fig 3(d) where *ma* is small (∼ 0.25).

All these results can be understood by the geometrical characteristics of the networks. Initially, the density of neighboring MTs (MTs that are within the reach of the motors from the cargo) is higher in the QRS, decreasing later (∼ 1/*r*) whereas in the NRS is approximately uniform. This uniformity is due to the fact that the track crossings are distributed along the whole network. In the QRS, the crossings occurs mainly in the initial core of the structure. Consequently, if a motor detaches from one MT, it is more likely to attach to another MT in NRS than in QRS. However, globally, the motors seem to be organized by different tracks and although in the QRS (with 80 MTs) the correlation in the same MT is increasing, this effect, as we will see later, is more evident with a smaller number of MTs.

We have highlighted two crucial facts. First, active transport is faster in NRS and is more likely to occur along different MTs but with a gradual increasing of the organization for the QRS. Second, in both networks, motor interaction is predominantly collaborative. Both facts are due to the spatial arrangement of the MTs in both networks.

#### Influence of number of microtubules in cargo-motor dynamics for two kinesins in the NRS and QRS configurations

The density of the MTs and the crossing points between the tracks depend on the number of MTs. Therefore, it would be interesting to know if transport is efficient for any number of MTs in networks like NRS and QRS. It is expected that the variability of MTs disrupts motor organization and thus the interaction between kinesins. Due to network characteristics, the influence of number of MTs should be more important in NRS than in QRS (see Fig 1).

**Fig 4:**
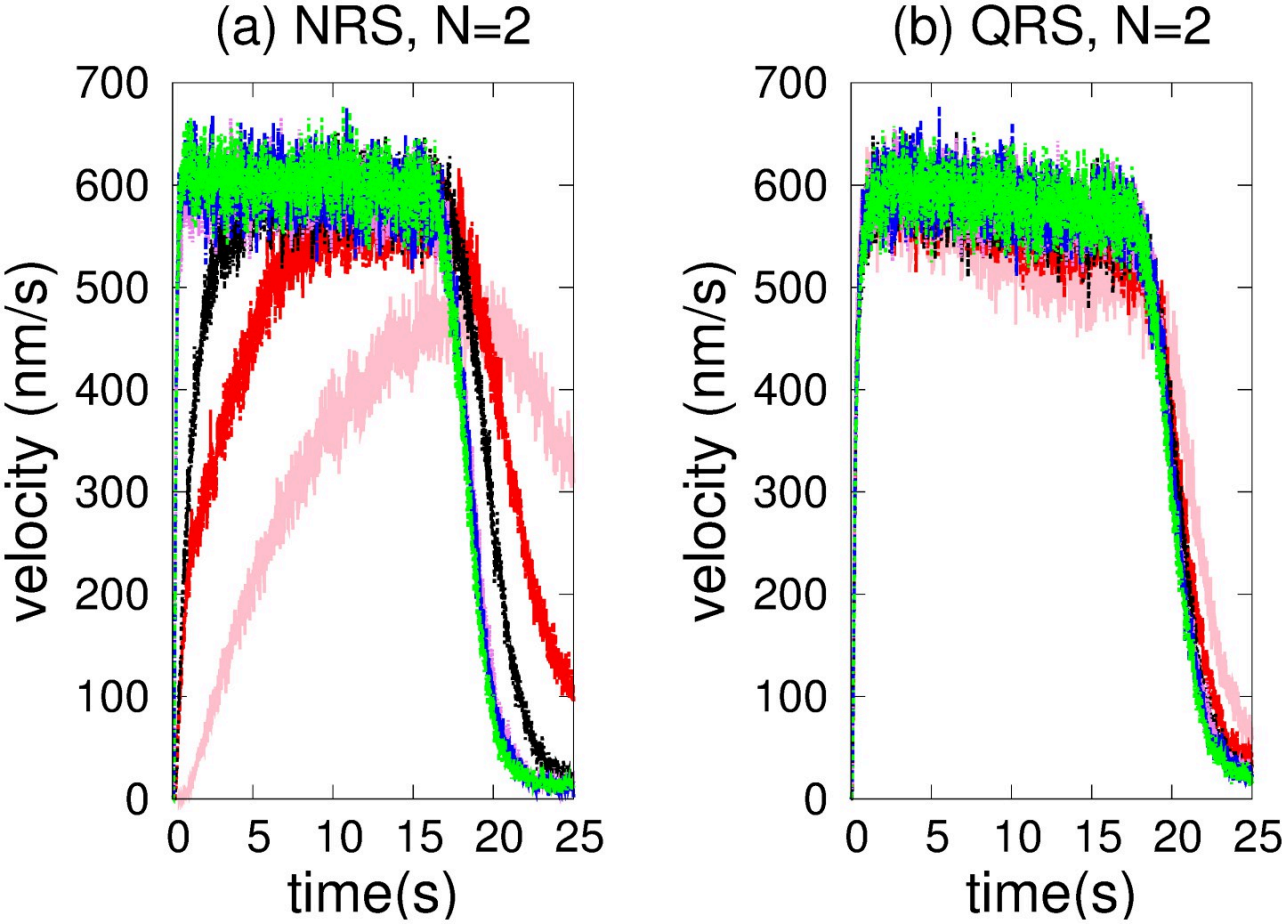
Movement of two kinesins in the NRS and QRS networks with varying numbers of MTs. The number of MTs is indicated by colors written between parentheses: 20 (pink), 40 (red), 60 (black), 70 (violet), 80 (blue) and 100 (green).(a) NRS. (b) QRS.

**Fig 5:**
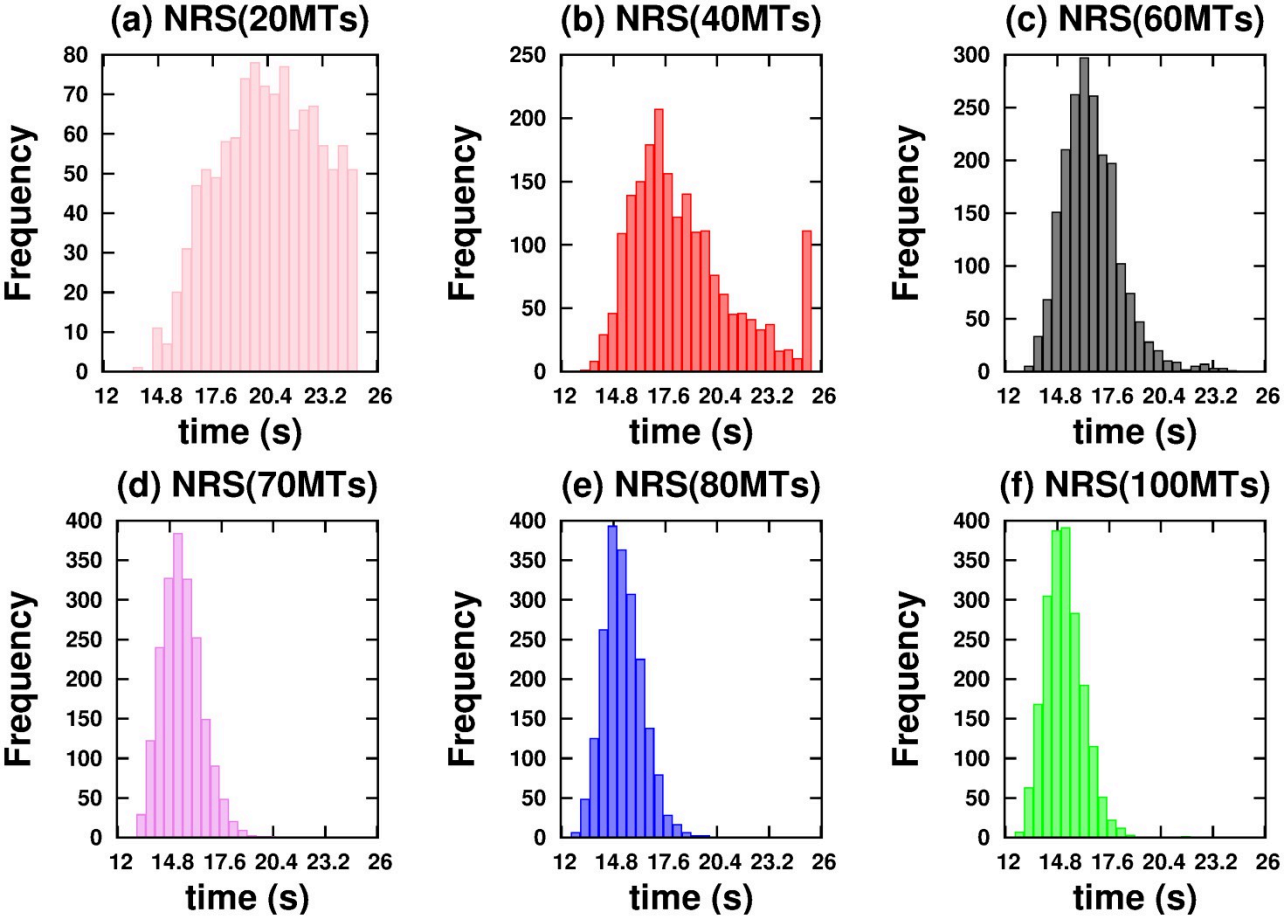
Histograms of the first passage times to reach a distance of 10000 *nm* on the *NRS* configuration. Cargo is driven by two kinesins. (a) 20 MTs. (b) 40 MTs. (c) 60 MTs. (d) 70 MTs. (e) 80 MTs. (f) 100 MTs.

**Fig 6:**
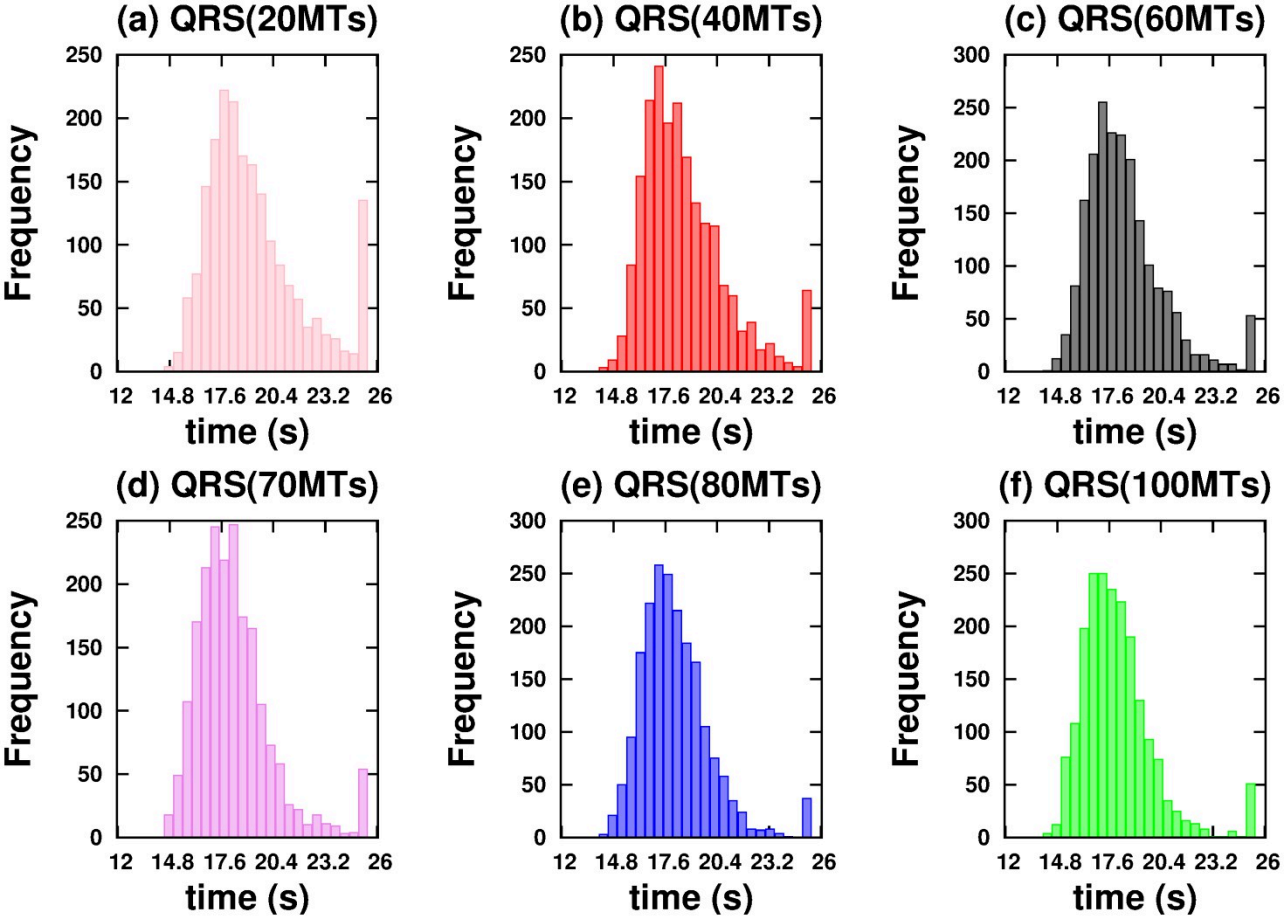
Histograms of the first passage times to reach a distance of 10000 *nm* on the QRS configuration. Cargo is driven by two kinesins. (a) 20 MTs. (b) 40 MTs. (c) 60 MTs. (d) 70 MTs. (e) 80 MTs. (f) 100 MTs.

Fig 4 shows *vc* as a function of time for NRS and QRS. In both networks, we considered a variable number of MTs. It should be noted that only in the NRS does the *vc* change significantly for number of MTs less than a critical number of 70. Below this number, the *hfcpt* show significant differences: wider distributions and a large asymmetry towards slow trajectories (see Fig 5). For example, the mean, mode, standard deviation, and skewness coefficient values for 20 MTs are 77.78 *s*, 19.7 *s*, 64.38 *s*, and 0.23, respectively (refer to S1 Appendix). Therefore, the significant right-hand skewness suggests that a considerable number of trajectories require over 25 *s* to reach 10000 *nm*. With an increase in the number of MTs, the dispersion and the differences between means and modes decrease, resulting in less asymmetric distributions (refer to the values in the S1 Appendix). However, in the QRS, *hfcpt* show very little difference as the number of MTs changes (see Fig 6) and the values of the means, modes, standard deviations and skewness coefficients do not show significant differences (see values in the S1 Appendix). The reasons for these behaviours are explained below.

**Fig 7:**
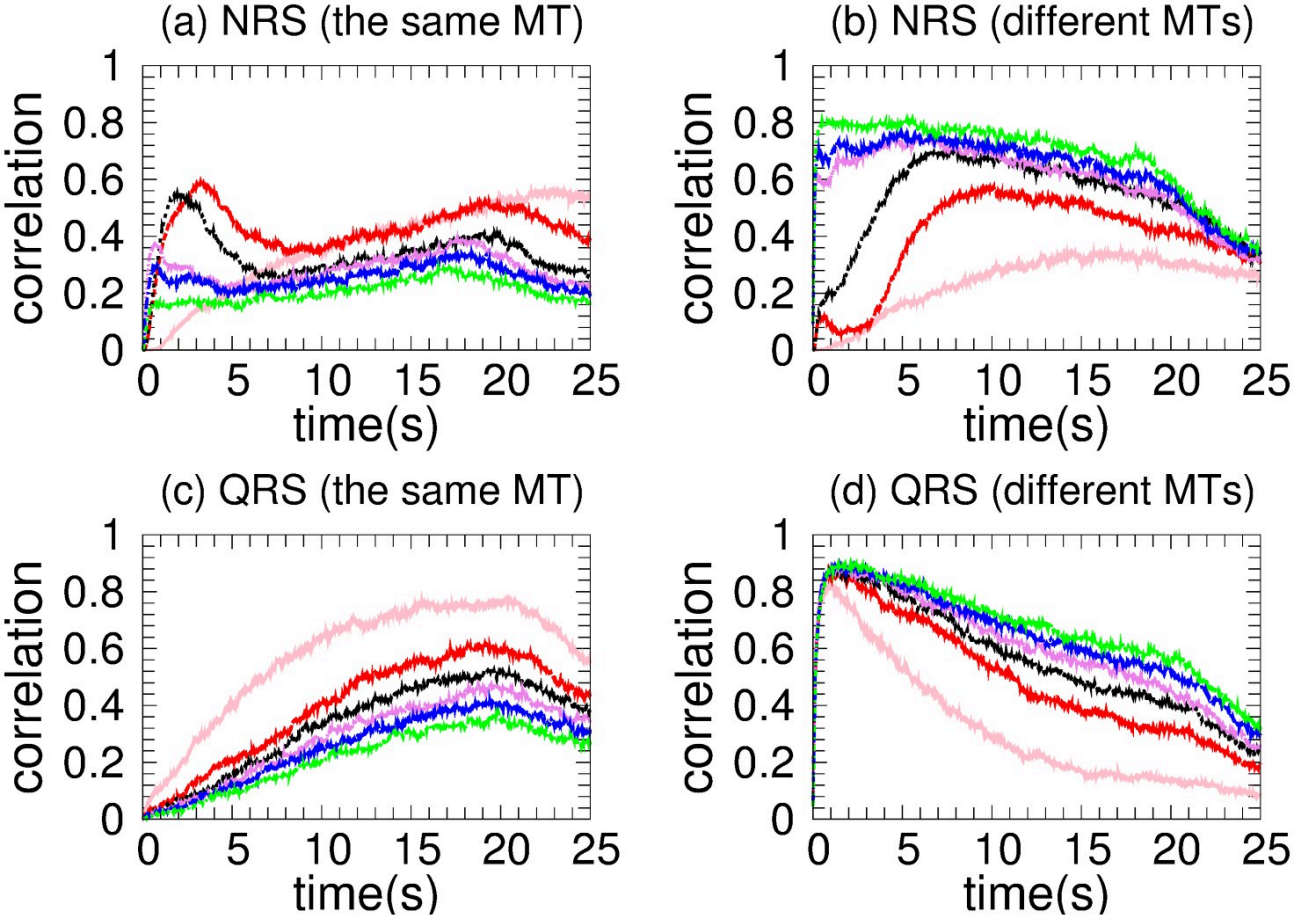
Motor correlation of two kinesins for different numbers of MTs which are identified with the same colors in Fig 4. (a) and (c) are devoted to correlation along the same MT in the NRS and QRS, respectively. (b) and (d) are devoted to correlation along different MTs in the NRS and QRS, respectively.

**Fig 8:**
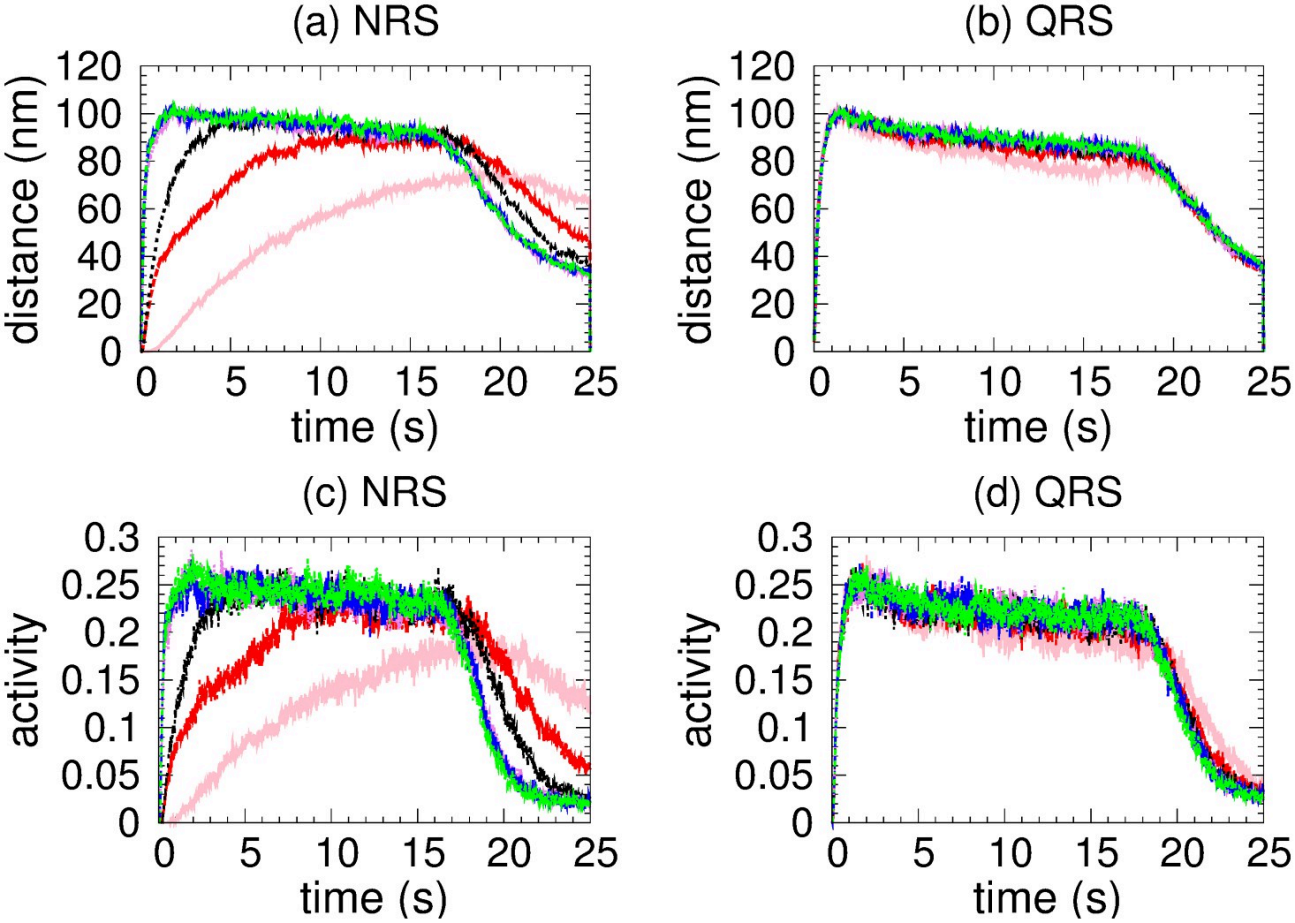
Mean intermotor distance. ((a) NRS and (b) QRS) and motor activity ((c) NRS and (d) QRS) for different number of MTs. The colors identifying the number of MTs are the same as in Fig 4.

In an NRS, for 70 MTs and above, the density of MTs and their crossover points are high enough (see Fig 1) to allow motors to move in different MTs (see Fig 7) without competition and thus high velocities (high *vc*). This is also observed in the correlations (see Fig 7) Since *mc*, *ma* has small values (see Fig 8), we deduce that at least one kinesin is pulling the cargo. Because all trajectories used in transport are fast (see *hfcpt* in Fig 5) and *mid* takes intermediate values (see Fig 8), motor interaction must be cooperative and according to the *vc* (see Fig 4), the transport is efficient.

However, for 40 and 60 MTs, kinesins are more likely to walk on the same MT in the early moments due to the lower density of MTs. Then, this probability decreases as the probability of walking through different MTs increases (see Fig 7). This is due to increased density and crossing points (see Fig 1). In Fig 5(a)–5(c), we clearly observe that slow trajectories contribute decisively to the average velocity. On the other hand, the *ma* is very small as well as the *mid* (see Fig 8). All these results shows that, with 40 and 60 MTs, there is a large number of trajectories in which motor interaction is competitive, mainly by the interaction of the cargo with motors in different MTs and reduces the transport efficiency. This competitiveness is amply demonstrated in the network with 20 microtubules, where many trajectories are inefficient for transport.

On the other hand, in QRS, the *vc* profile is almost insensitive to the number of MTs. In the beginning, kinesins are more likely to walk in different MTs (high *mc*) due to their high density. As the density begins to decrease (see Fig 1), so does the *mc* (for different MTs), while the *mc* for the same MT gradually increases (see Fig 7). These behaviors are more noticeable when the number of MTs is less than 70. In these cases, the *mc* for the same MT may be significant. However, there is no significant change in *vc* even though some differences in the histograms are observed (see Fig 6). In addition, the *vc* values indicate the transport efficiency for any number of MTs and *mc*. The insensitivity of the *vc* is possible because at least one kinesin is pulling the cargo. Since *mid* and *ma* have medium and low values, respectively, the motor interaction must be non-competitive as a single motor is the main responsible of the transport. A similar situation is verified for small and very small values of *mid* and *ma*, respectively.

In conclusion, we have found that only NRS has a critical number of MTs (70) below which the *vc* is sensitive when the number of tracks changes. For 40 and 60 MTs, motor interaction may change the regime and transport may be less efficient than that of high density MTs.

#### The influence of motor detach probabilities in cargo velocities in NRS and QRS configuration

Since many aspects of the observed phenomenology are grounded on motor attaching and detaching events, we study the effect of the detaching probability. We expect to find an optimal range of detachment probability for which the velocity reaches the maximum value. For high detachment, the transport is inefficient since motor easily unbinds from MTs, providing low motor activity and hence low velocity. On the other hand, a low detachment probability impedes motor organization in the same MT (low correlation) and favors competition of motors walking on different MTs.

**Fig 9:**
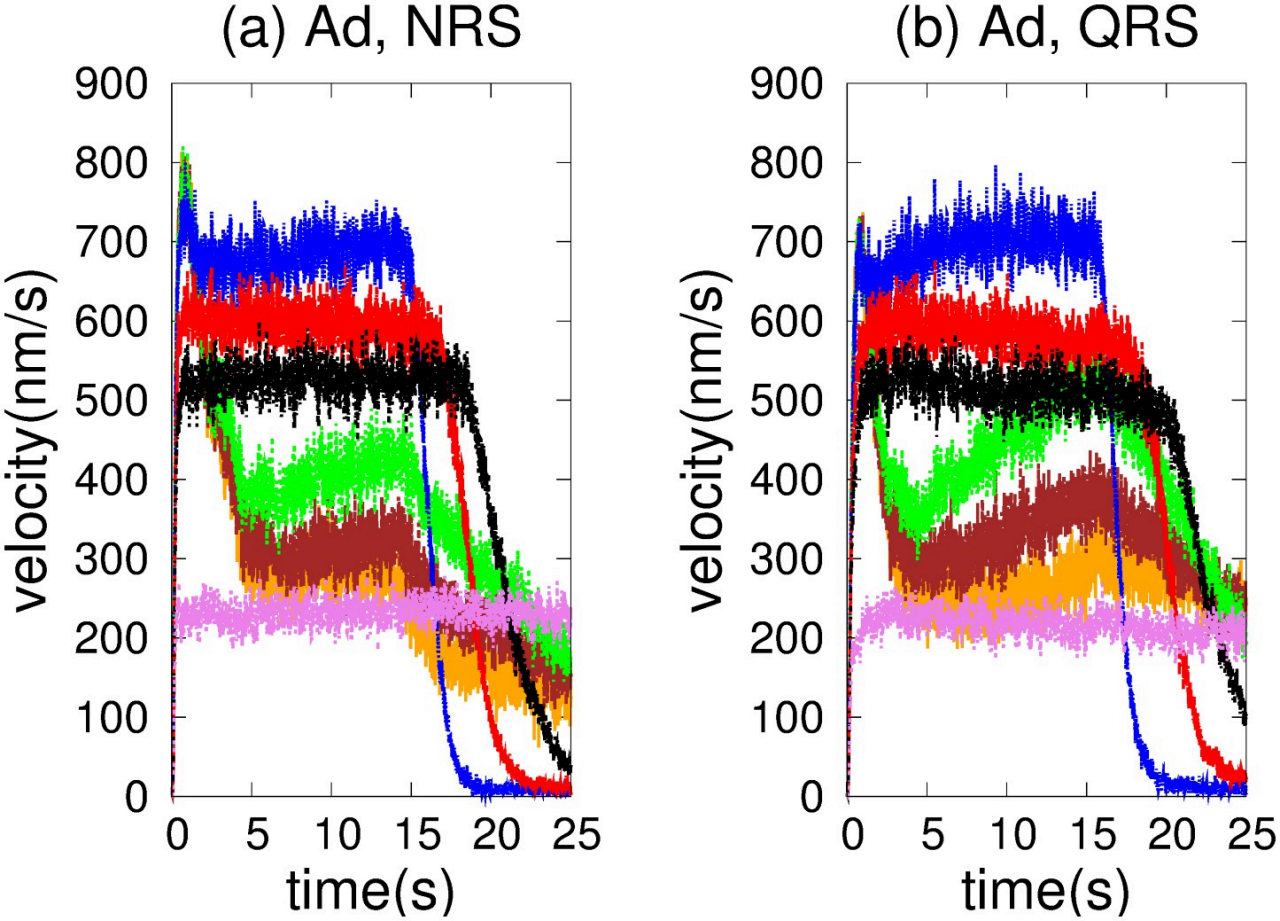
Cargo velocity for different coefficients *Ad* of the detachment rate. Let us assign the orange color to *Ad* = 0.002; brown color, *Ad* = 0.004; green color, *Ad* = 0.01; blue color, *Ad* = 0.2; red color, *Ad* = 1.0; black color, *Ad* = 1.5, and violet color, *Ad* = 5.0. With 80 MT: (a) NRS configuration. (b) QRS configuration.

**Fig 10:**
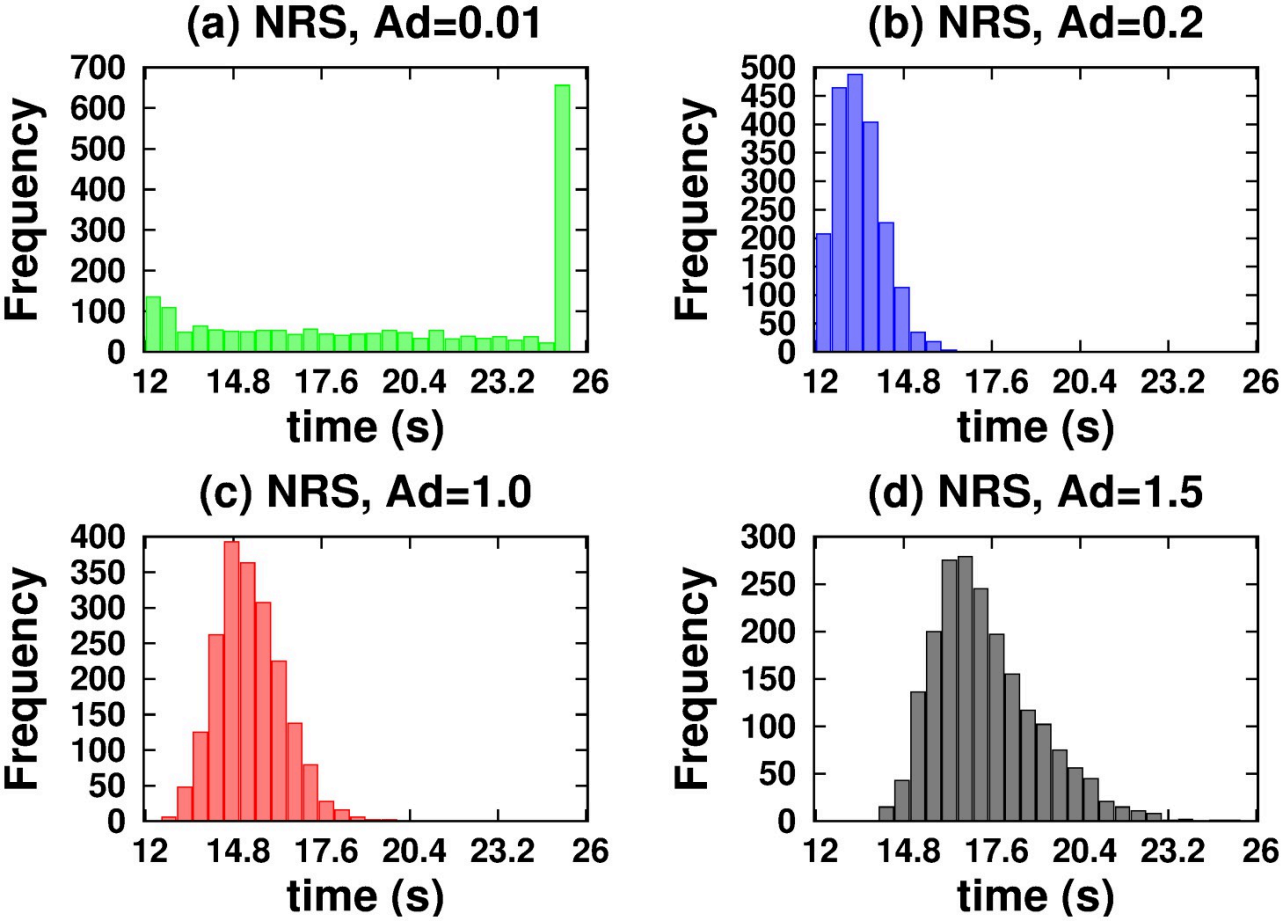
Histograms of the first cargo passage times to reach a distance of 10000 *nm* when it is driven by two kinesins. For the NRS configuration: (a) *Ad* = 0.01. (b) *Ad* = 0.2. (c) *Ad* = 1.0. (d) *Ad* = 1.5.

**Fig 11:**
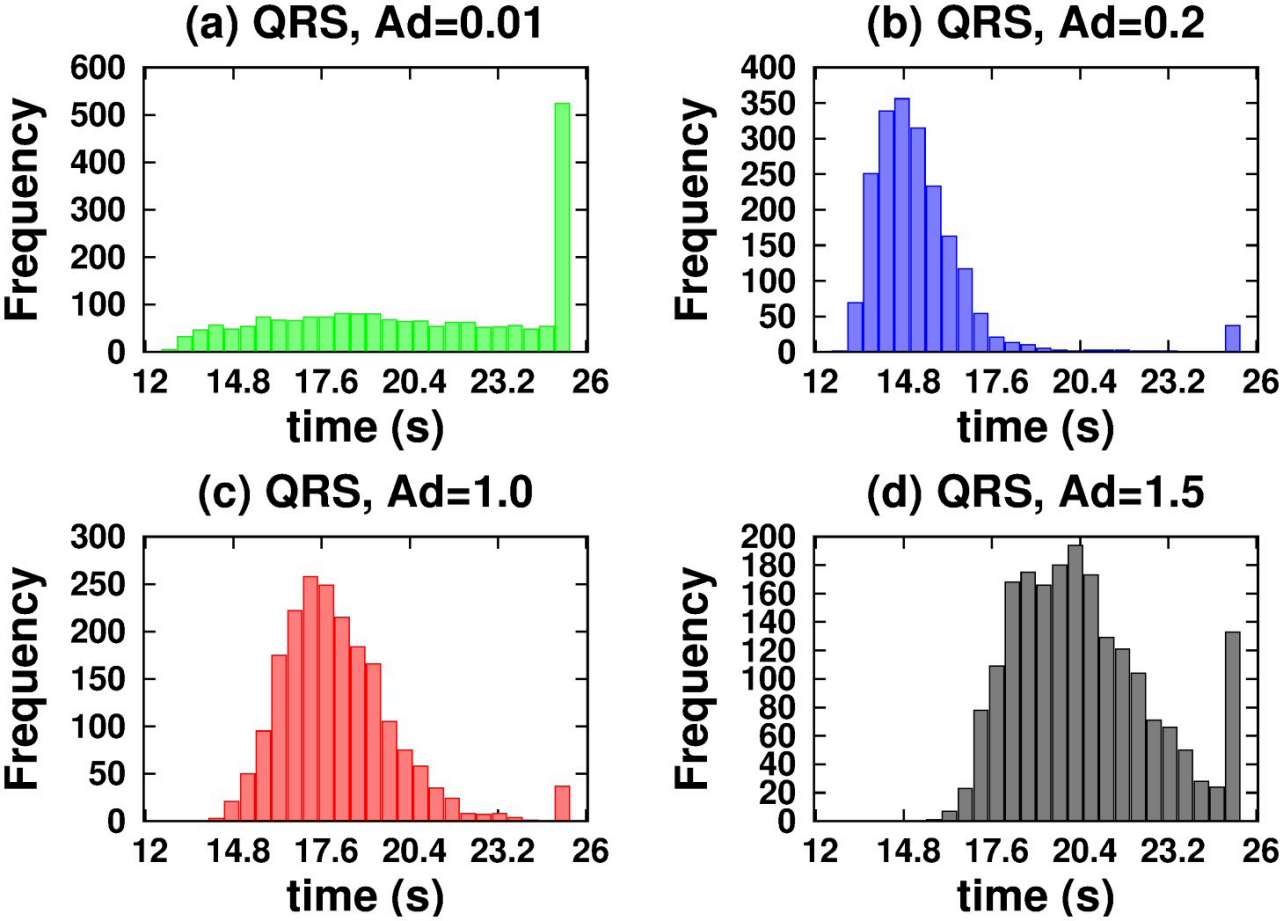
Histograms of the first cargo passage times to reach a distance of 10000 *nm* when it is driven by two kinesins. For the QRS configuration: (a) *Ad* = 0.01. (b) *Ad* = 0.2. (c) *Ad* = 1.0. (d) *Ad* = 1.5.

**Fig 12:**
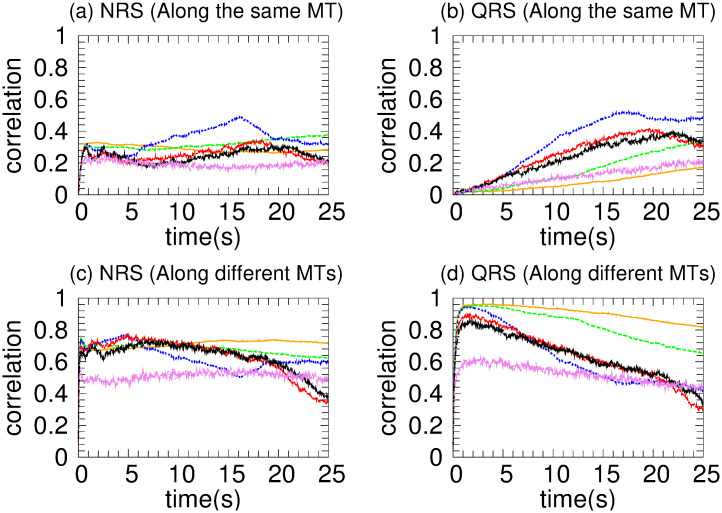
Motor correlation of two kinesins for different detachment coefficients and networks with 80 MT. These coefficients are identified with the same colors as in Fig 9. Correlations along the same MT are devoted for NRS and QRS in (a) and (b), respectively, whereas correlations along different MTs for NRS and QRS appear in (c) and (d), accordingly.

**Fig 13:**
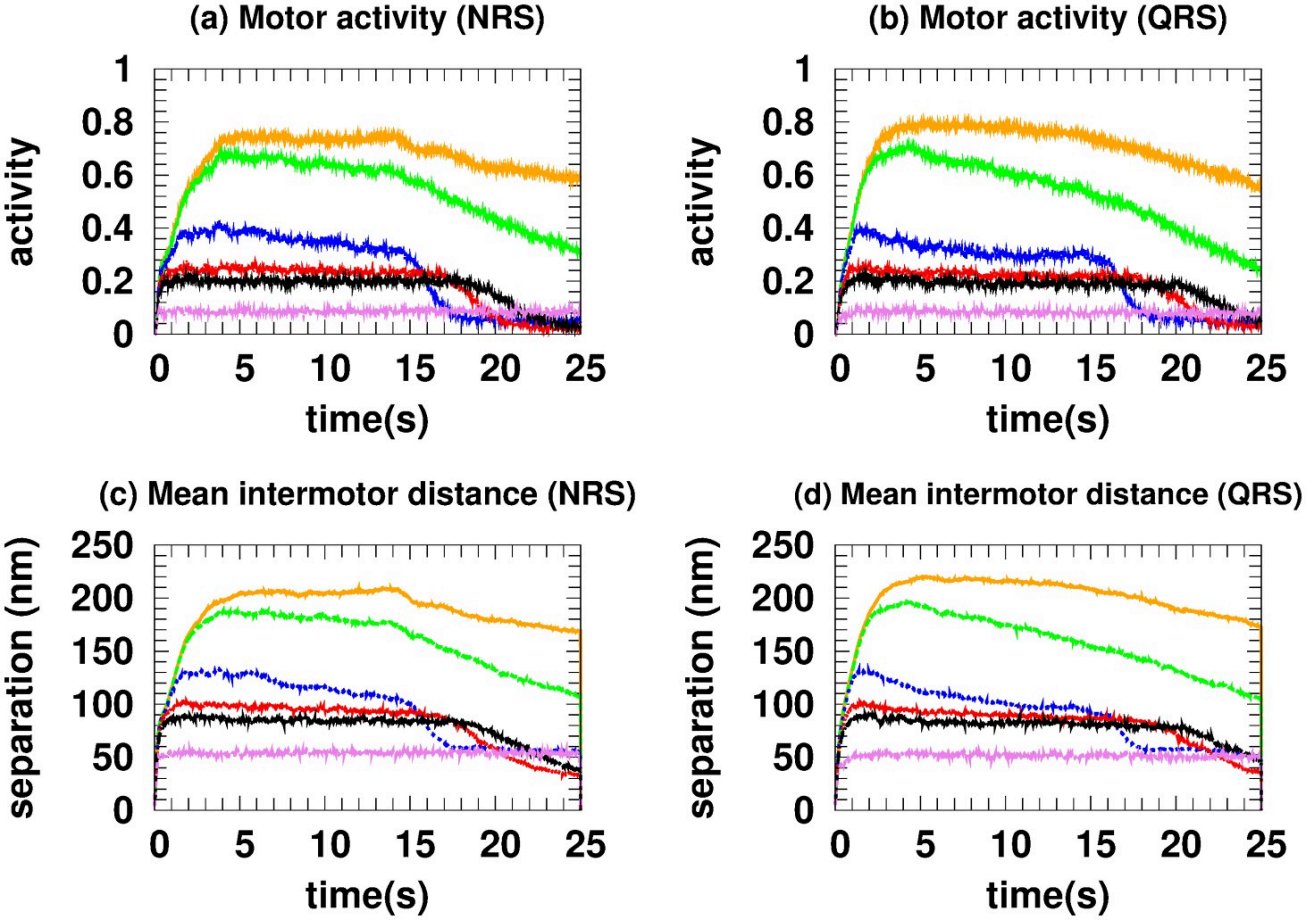
Mean intermotor distance. ((a) NRS and (b) QRS) and motor activity ((c) NRS and (d) QRS) for different detachment coefficients whose values are identified with the same colors as in Fig 9.

Fig 9 displays *vc* as a function of time for various coefficients of the detachment rate *A*_*d*_. We have taken 80 MTs in both NRS and QRS settings. The *vc* is higher for *A*_*d*_ values in the range of [0.1 − 1.0] and quickly decreases out of this range. For very low values of the detachment rate (*A*_*d*_ = 0.002), a fast increase of the velocity over time is observed, as consequence of the slow increase of motor organization followed by a gradual decay until a stationary regime is achieved (around 5 s). This decay is due by the competence between motor walking in different MTs that cannot been organised through unbinding-binding events. However, this phenomenon does not occur at high unbinding rates because the organization is disrupted in every time by the frequent unbinding of motors. In order to understand these behaviors, we performed a joint analysis of the quantities shown in Figs 10–13.

In the NRS, in [0.1 − 1.0], transport is efficient because it occurs along fast trajectories (see the *hfcpt* in Fig 10) with distributions that have very small differences between means and modes, and whose skewness profiles contain fast trajectories (consult the values presented in the S1 Appendix). This is possible because even though the kinesins are organized by different MTs (see Fig 12) and have low *ma* (see Fig 13), at least one kinesin is pulling the cargo without motor competition. This conclusion is consistent with *mid* values (see Fig 13). In the QRS, the transport behavior is similar to that of the NRS. However, some differences can be observed. These include a few slow trajectories and quantities such as the following *mc*, *ma*, and *mid* (see Figs 11–13). For both NRS and QRS, transport is most efficient (higher *vc*) when *A*_*d*_ = 0.2 and motor interaction is cooperative. Moreover, for any value of *Ad* in [0.1–1.0], transport is more efficient in the NRS than in the QRS.

## Conclusions

In this work we have analyzed the active transport of the cargo driven by two kinesins (considered as particles) moving over MTs located in 2-D networks such as NRS and QRS. The MTs are considered straight lines (see Fig 1), with the same polarity (minus-plus), with different spatial orientations (quasi-radial and non-radial) and with crossings with other MTs that could change the direction of the transport [29, 41], as the motors may interact with multiple MTs simultaneously at intersections or because they stochastically detach and rebind to a different MTs [31, 32]. The first option is similar to the one reported in the reference [30] resulting in a motor interaction whose character is mostly cooperative or competitive or both coexist along a given trajectory.

Theoretically, our model shares common features with other models reported in the literature [19, 34, 37]. Molecular motors can detach from the tracks and reattach to them and can occupy discrete positions along the MTs located in the 2-D region. The interaction between kinesins is modeled by including in the Monte Carlo algorithm a constraint that prohibits two motors from being in the same position. The kinesin can perform forward and backward steps. If a kinesin does not detach from a given MT, it continues to walk along the same MT or another one that intercepts it. We have considered the cargo as a particle performing a continuous overdamped Brownian motion in 2 dimensions. The cargo is attached to each motor by a nonlinear spring [34]. Although our model is comparable to others previously used, the inclusion of multiple MT as tracks for motors introduces a new variable. In order to keep the model as simple as possible, this MT network is modeled as strait lines without volume which allow modifications both in geometry and density (although these could be not realistic).

As a consequence of the unsynchronized stepping of the kinesins, forces on the cargo and correlations between the motors are generated [18]. Although we have not studied a gliding assay, these facts are very similar to those reported by the reference [39]. However, in our model the force is transmitted directly to the cargo by each motor and then, through the cargo, affects the dynamics of other kinesins. Thus, the *vc* is influenced by the collective dynamics of the motors, which in turn depends on the organization of the motors in the MTs configuration. Therefore, we have analyzed the collective dynamics of the system (motors and cargo) with the *vc*, *hfcpt* and statistical variables (mean, mode, standard deviation and skewness), the *mid*, *mc* and *ma*. These magnitudes have been calculated taking into account the NRS and QRS configurations (see Fig 1), the number of MTs and the motor detach probabilities. As a result, we have observed a complex dynamics which which is in agreement with that observed in reference [33–35]. The motion of cargo is determined by the geometric characteristics of the networks and it is a function of the neighboring MTs [33] and their size (in our case 12000 nm) [34, 35]. The joint analysis of our defined magnitudes allows us to deduce if the interaction between two kinesins is collaborative, competitive and with coexistence of both (in [39] the mechanisms of cooperativity for a pair of molecular motors is discussed).

Finally, we summarise the main results of our research.

Active transport is more efficient in NRS than in QRS. Almost all trajectories are fast in NRS, but not in QRS (see Figs 1 and 2) Showing different regimes of competitiveness.

For any number of MTs, in NRS and QRS, kinesins are more likely to advance through different MTs (see motor correlation defined by us and Figs 3 and 7). Consequently, excluded volume effects are less likely to occur in the motor interaction.

Only in an NRS there is a critical number of MTs (70) below which the dynamics of the cargo is very sensitive (see Fig 4). Transport is not very efficient for 40 and 60 MTs and completely ineffective for 20 MTs. This fact does not occur in the QRS (for any number of MTs) and the NRS (with 70 or more MTs).

The *vc* is very sensitive with the detach rate (see Fig 9) and an efficient transport is only possible when this is in the range (0.1, 1). The nature of the interaction between two kinesins depends on their rate of detachment from the MTs and the structure of the network. A very strong competitive regime exists if the detachment rate is very large (*ma* and *mid* decrease and *mc* has similar probabilities in the same or different MTs), which makes transport inefficient. On the other hand, a competitive regime exists if the detachment rate is very low (*ma*, *mid* and *mc* by different MTs increase) which causes the speed to decrease in a very short time and the transport slows down. However, if the detachment rate has a mean value in the interval ([0.01, 1.0]) the motor interaction is mainly cooperative for QRS with any number of MTs and NRS with 70 or more MTs.

## Supporting information

S1 Appendix Algorithms. (A) Acronyms of physical quantities. (B) Values of Statistical quantities.Algorithms used for microtubule generation (C), crossing of microtubules (D) and (E) Monte Carlo for motion of molecular motors along the microtubules.
(PDF)
