# Supplementary material for: Organization of two kinesins in a two-dimensional microtubule network
Source: PLoS One. 2024 Mar 13;19(3):e0295652. doi: 10.1371/journal.pone.0295652 (PMC10936800; doi:10.1371/journal.pone.0295652)
Supplement: S1 Appendix — (A) Acronyms of physical quantities. (B) Values of Statistical quantities.Algorithms used for microtubule generation (C), crossing of microtubules (D) and (E) Monte Carlo for motion of molecular motors along the microtubules. (PDF) [file pone.0295652.s001.pdf]

SUPPORTING INFORMATION: S1 APPENDIX
Organization of two kinesins in a two-dimensional microtubule network

J. M. Bergues and F. Falo

A ACRONYMS OF SOME PHYSICAL QUANTITIES

Magnitude	Acronyms
velocity of the cargo	*vc*
histograms of the first cargo passage times	*hfcpt*
motor correlations	*mc*
motor activity	*ma*
mean intermotor distance	*mid*

B VALUES OF STATISTICAL QUANTITIES

NRS, N=2, Ad=1, Fd=3N, Fs=6N

MTs	Mean	St. deviation	Skewness	Mode
20	77.78251	64.38324	0.2287530	19,7
40	18.55874	2.728437	0.8123671	17.4
60	16.63602	1.550808	0.9550782	16.2
70	15.45241	1.071084	0.4835318	15.1
80	15.32727	1.044395	0.4701973	14.5
100	15.15976	1.016767	0.5601879	15.1

QRS, N=2, Ad=1, Fd=3N, Fs=6N

MTs	Mean	St. deviation	Skewness	Mode
20	19.31610	2.463526	0.8710376	17.9
40	18.60073	2.181456	1.045675	17.4
60	18.40435	2.025142	1.187730	17.4
70	18.24855	2.018148	1.279762	18.4
80	18.12516	1.846503	1.174034	17.2
100	18.01251	1.923197	1.417311	16.9, 17.4

N=2, 80 MTs, Fd=3N, Fs=6N

Network/Ad	Mean	St. deviation	Skewness	Mode
NRS/0.01	19.71385	4.867866	-0.2492299	≥ 25
NRS/0.2	13.37275	0.7861902	0.6041790	13.25
NRS/1.0	15.32727	1.044395	0.4701973	14.5
NRS/1.5	17.40001	1.647412	0.8453217	16.85
QRS/0.01	20.57162	3.819342	-0.2622937	≥ 25
QRS/0.2	15.32055	1.801505	3.196247	14.6
QRS/1.0	18.12516	1.846503	1.174034	17.2
QRS/1.5	20.52108	2.164449	0.4843402	20.2

C MICROTUBULE GENERATION

For each MT:

- we randomly compute the coordinates x_0 and y_0 (negative end of MT).
- we randomly calculate the orientation angle of the MTs;

- we determine x and y coordinates (positive end of MT). We add up to x_0, (y_0) the length of Mt multiplied by the cosine (sine) of the orientation angle;
- we find out if MT intercepts the borders of the region;

1. if MT intercepts the boundary, the MT is rejected and process is repeated again.
2. if the MT does not intersect the borders, the MT is considered and algorithm goes to generation of next MT.

D MICROTUBULE INTERSECTION POINTS

For each pair of MTs

- we calculate the sites corresponding to each MT at which the intersection occurs.
- we compute x and y coordinates of the intersection point of each MT in terms of their respective sites of intersection previously determined;
- we find out if the x and y are greater than the initial coordinates and less than the final ones. Then,
    1. If x and y do not satisfy the previous condition, algorithm goes to prove other pair of MT.
    2. If x and y satisfy the set condition, the sites are approved.
- if the sites are accepted, we will generate a matrix. Each row represents the number of a MT. Each column indicates the number of the site at which a MT is intercepted with other.

E MONTE CARLO ALGORITHM WITH FORWARD AND BACKWARD STEPS FOR INTERACTING MOTORS

This algorithm is a modified version of algorithm developed in reference [1]. In this work, it has been extended to two dimensions and an arbitrary number of microtubules. At each time step:

1. For each attached motor;
    - we compute the force f_i depending on $(r_i - r_c)$;
    - motor i detaches with probability $P_{\text{det}}(f_i)$. The position of the motor is set to that of the cargo and the algorithm moves on to the next motor.
    - if the motor does not detach, then
        - we compute the probability $1 - dt/\tau_D(f_i)$ of staying in a site and evaluate the possibility of staying;
        - if the staying is approved, the new position of motor is equal to the previous one and algorithm goes to the next motor;

- if the staying is not approved, then
  (a) it is assigned to perform jumps either forward or backward with probability P_f or P_b , respectively. These movements either might occur by the same MT or other. And,
  (b) the forward (backward) jump is performed only in case the right (left) site is empty. The new position will be considered for computing f_i in the next time step. If the right (left) site is not empty nothing happens and we go to the next motor.

2. With probability $\Pi\ dt$ each detached motor attaches to a random empty site located in the interval $|r - r_c| < r_0$.

3. The cargo advances accordingly to the Langevin dynamics for a time interval dt with $f = \sum_i f_i$ (A standard stochastic Euler algorithm is considered for the integration). It is calculated velocity and cargo positions respect to coordinate origin.
   - if $f_i > 0$, motor i is a pulling motor;
   - if $f_i = 0$, motor i is not a pulling motor and algorithm goes to the next motor.

References

[1] S. Bouzat and F. Falo, The influence of direct motor-motor interaction in models for cargo transport by a single team of motors, Phys. Biol. **7**, 046009 (2010).
